# Awareness of Aging Mediates the Relationship Between Health and Control Beliefs: A Micro-Longitudinal Study

**DOI:** 10.1093/geroni/igaa057.1449

**Published:** 2020-12-16

**Authors:** Shenghao Zhang, Shevaun Neupert

**Affiliations:** North Carolina State University, Raleigh, North Carolina, United States

## Abstract

Objective: Control beliefs are bidirectionally related to physical and cognitive health, but it is unclear how health influences control beliefs. Health-related experiences (physical symptoms and memory failures) on a particular day can make older adults more aware of their aging, and may subsequently lead to lower control beliefs. We propose that awareness of aging constructs (subjective age and awareness of age-related change [AARC]) could function as mediating mechanisms between health and control beliefs, and examine this relationship from both between- and within-person perspectives separately for domain-general and domain-specific control beliefs. Methods: Older adults (n=116) ranging in age from 60 to 90 (M=64.71) completed a nine-day daily diary study online, resulting in 743 total days. Participants reported their physical symptoms, memory failures, felt age, daily AARC gain and loss experiences, and control beliefs on Days 2-9. Results: Multilevel mediation results showed that between-person AARC losses mediated the relationship between physical symptoms and both domain-general and domain-specific control over physical symptoms. Between-person AARC losses also mediated the relationship between memory failures and both domain-general and domain-specific control over memory. AARC gains and subjective age did not mediate the relationship between health and control beliefs. Discussion: Our findings suggest that between-person differences in AARC losses function as underlying mechanisms linking health and control beliefs. Efforts to reduce AARC losses may lessen the negative impact of health problems on control beliefs for older adults.

